# Marital Status, Lifestyle and Dementia: A Nationwide Survey in Taiwan

**DOI:** 10.1371/journal.pone.0139154

**Published:** 2015-09-28

**Authors:** Ling-Yun Fan, Yu Sun, Huey-Jane Lee, Shu-Chien Yang, Ta-Fu Chen, Ker-Neng Lin, Chung-Chi Lin, Pei-Ning Wang, Li-Yu Tang, Ming-Jang Chiu

**Affiliations:** 1 Department of Neurology, En Chu Kong Hospital, New Taipei City, Taiwan; 2 Department of Neurology, National Taiwan University Hospital, Taipei, Taiwan; 3 Taiwan Alzheimer’s Disease Association, Taipei, Taiwan; 4 Department of Neurology, Taipei Veterans General Hospital, Taipei, Taiwan; 5 Department of Computer Science and Information Engineering, Chung Gung University, Tao-Yuan, Taiwan; 6 Department of Neurology, National Taiwan University Hospital, College of Medicine, National Taiwan University, Taipei, Taiwan; 7 Graduate Institute of Brain and Mind Sciences, College of Medicine, College of Medicine, National Taiwan University, Taipei, Taiwan; 8 Graduate Institute of Psychology, College of Science, National Taiwan University, Taipei, Taiwan; 9 Graduate Institute of Biomedical Engineering and Bioinformatics, College of Electric Engineering and Computer Science, National Taiwan University, Taipei, Taiwan; University of Utah, UNITED STATES

## Abstract

**Background:**

Evidence of an association between lifestyle and marital status and risk of dementia is limited in Asia.

**Methods:**

In this nationwide population-based cross-sectional survey, participants were selected by computerized random sampling from all 19 counties in Taiwan. A total of 10432 residents were assessed by a door-to-door in-person survey, among whom 7035 were normal and 929 were diagnosed with dementia using the criteria recommended by National Institute on Aging-Alzheimer’s Association. Premorbid lifestyle habits and demographic data including marital status were compared between normal subjects and participants with dementia.

**Results:**

After adjustment for age, gender, education, body mass index, smoking, drinking, marital status, sleep habits, exercise, social engagement and co-morbidities including hypertension, diabetes and cerebrovascular diseases, an increased risk for dementia was found in people with widow or widower status (OR 1.42, 95% CI 1.15–1.77) and people who used to take a nap in the afternoon (OR 1.33, 95% CI 1.02–1.72). Decreased risk was found in people with the habit of regular exercise (OR 0.12, 95% CI 0.09–0.16), adequate night sleep (OR 0.55, 95% CI 0.39–0.76) and regular social engagement (OR 0.53, 95% CI 0.36–0.77).

**Conclusions:**

Our results provide preliminary evidence of possible risk-reduction effects for dementia, including regular exercise even in modest amounts, social engagement and adequate night sleep, whereas people with the widow/widower status or who used to take an afternoon nap might have increased risk of dementia.

## Introduction

Previous studies in Taiwan reported that the prevalence of dementia in adults aged over 65 ranged between 1.7% and 4.3% based on surveys of local urban or rural areas.[1–4] The pooled prevalence between 1991 and 1994 was approximately 3.6% based on 11,208 study subjects.[5] In a most recent nationwide survey (2011–2012) on Taiwanese population aged 65 years and above, the age-adjusted prevalence of all-cause dementia was 8.04%, which is the data basis of this report.[6]

Many risk factors of dementia, such as vascular issues, metabolic problems, dietary habits, and genetic factors, have been proposed.[7] According to the concept of brain reserve [8], people with greater brain reserve can tolerate more severe pathological change until the clinical manifestation of apparent cognitive decline. Therefore, concepts to identify the protective or modifiable factors are warranted. Lifestyle factors such as exercise and social engagement are under epidemiological study evaluation and are also targets in intervention studies for delaying cognitive decline.[9]

Additionally, genetic and environmental factors could interact and modify the risk of dementia; therefore, it is worthwhile to evaluate the association or risk factors of developing dementia in each ethnic group in various regions worldwide. The aim of our study is to investigate the association between lifestyle factors, including sleep and marital status, and dementia among people aged 65 years and older in Taiwan.

## Methods

The study has been approved by the National Taiwan University Hospital's Institutional Review Board. All investigation has been conducted according to the principles expressed in the Declaration of Helsinki. We obtained Written Informed Consent from the participants or their proxy.

### Study design

This study is a nationwide population-based case-control investigation. All 10432 participants aged 65 years and older were randomly sampled and enrolled from December 2011 to March 2013. They completed the survey using door-to-door screenings.[6] After obtaining informed consent, we performed in-person interviews to collect a brief history focusing on cognitive and functional status, followed by a structured questionnaire recording demographic data and lifestyle factors, as well as mental tests. The interview process was performed according to an operational manual that defines all variables examined in this questionnaire. Logic checks for inconsistency and auditing were performed by experienced supervisors to ensure the quality and reliability of the entered data.[6] A medical history was taken to detect any insidious change of behavior or personality and any mental decline from previous levels of functioning.[6] The diagnosis of dementia, particularly difficult cases, was re-examined and discussed by a consultant panel consisting of four neurologists and one clinical psychologist specialized in the diagnosis and management of patients with dementia. The sampling process, training of interviewers, home visiting procedure, quality control, and protocol approvals were detailed in our previous report. All interviews were conducted by well-trained field interviewers with continuous quality control to achieve necessary quality standards. The inter-rater reliability of global CDR was substantial, with a kappa value of 0.671.[6] The participation rate of this original nationwide survey was 36.5%.[6]

### Dementia diagnostic procedures

The diagnosis for all-cause dementia was based on the core clinical criteria recommended by the National Institute on Aging-Alzheimer’s Association (NIA-AA). Cognitive and functional status were determined from the evaluation with the participant or a knowledgeable informant who had taken care of the people with dementia for more than 10 hours a week and were capable of detecting insidious changes in behavior, personality, decline in mentality or function at work or activities of daily living (ADL). Objective assessments included the Clinical Dementia Rating Scale (CDR) and the Taiwanese Mental State Evaluation (TMSE). Normal TMSE results were defined as a score >24 in literate elders and >13 in illiterate elders.[10] Participants who were compatible with the NIA-AA criteria for all-cause dementia with impairment in 2 or more cognitive domains as well as a decline in daily functions, whereby the cognitive deficits were sufficient to interfere with independence of daily living function as a result of abnormality in community affairs or at-home hobbies or as a result of personal care as assessed by the CDR. Functional status was assessed using the ADL scale and the instrumental activities of daily living (IADL) scale. People with major depression, other mental disorders, delirium or other serious physical problems leading to cognitive or functional status impairment did not fulfill the NIA-AA criteria for all-cause dementia and were therefore excluded.

### Definition of the variables of lifestyle habits and marital status

During the interview, lifestyle habits, including social activity, regular exercise, smoking, drinking, chewing betel nuts, sleep habits and marital status, were recorded in detail. Information about the duration (years) of the habits was also recorded. Those habits should be developed before the onset of dementia.

#### Regular exercise

Exercise was defined as physical activities persisting for at least 20 minutes to an intensity capable of making one sweat. The exercise categories include swimming, mountain climbing, jogging, exercise walking, dancing and others. Participants were divided into three subgroups. “Regular exercise” indicated the frequency was at least once per week, whereas “exercise sometimes” indicated less than once per week but at least once per month. “Never or rarely exercise” indicated the frequency was less than once per month.

#### Social activities

“Active and regular social activity” occurred at least once per week, including attending clubs or social groups, engagement in religious activities, meeting friends and family or others. “Social activities sometimes” indicated that these activities occurred less than once per week but at least once per month. “Never or rarely social activities” indicated that these activities occurred less than once per month.

#### Smoking, chewing betal nut and drinking

These activities were considered habits if they occurred three or more times per week. Additionally, information including the type of alcoholic drink (liquor (whisky or sorghum), wine (red or white), beer, or rice wine) and drinking quantities per day were collected.

#### Night sleep

“Always good sleep” indicated that there were never any complaints of insomnia. “Occasional poor sleep” indicated that insomnia occurred one to three nights per week. “Often insomnia” indicated that insomnia occurred more than 3 nights per week, and “always insomnia” indicated that the interviewee could not fall asleep without hypnotics.

#### Afternoon nap

This lifestyle factor was considered to occur if the participant took a nap in the afternoon almost daily. Those who rarely took an afternoon nap were considered to not have this habit. The remaining participants were categorized as “taking nap sometimes”. We also asked them the average duration (hours and minutes) of the naps.

#### Marital status

Participants were categorized into “married and live with spouse”, “widow or widower” and “others”, which included divorced, unmarried, married but not living with spouse, or in a cohabitation status.

### Statistical Analyses

Categorical variables were represented by frequency or percentage, and the *x* ^2^ test was used for inter-block comparison. Means ± standard deviations were used for continuous variables, and the *t*-test was used for inter-block comparison. Univariate logistic regression analysis was used for to assess the association between all the aforementioned lifestyle variables and dementia and the calculation of crude OR and its 95% CI. Multiple logistic regression model 1 was adjusted for age, gender and education level. Model 2 represents model 1 plus adjustment for body mass index and comorbidities including hypertension, diabetes mellitus and history of cerebrovascular disease. Model 3 represents model 2 plus adjustment for marital status and lifestyle habits including smoking, drinking, chewing betel nuts, exercise, social engagement, night sleep, afternoon nap habit. We calculated the adjusted OR value and its 95% CI. We also tested the association between categorical variables by a contingency table. All analyses were performed using SAS statistical software (version 9.3) with 2-tailed statistical tests.

## Results

Among the 10432 participants, 929 cases (8.9%) fulfilled the NIA-AA core clinical criteria for all-cause dementia, whereas 7035 (67.4%) were diagnosed as non-demented, 2049 (19.6%) were classified with mild cognitive impairment and 419 (4.0%) participants were categorized in an unclassified group. The demographic data in the subjects with dementia (n = 929) and without dementia (n = 7035) are shown in Table 1.

**Table 1:** Demographic data of study participants (n = 7964).

	Non-demented (n = 7035)		Dementia (n = 929)	
**Continuous variables, n, %**	Mean	SD	Mean	SD
Age	74.89	6.03	81.71	7.43
Men	75.45	6.22	81.51	7.56
Women	74.30	5.78	81.82	7.35
**Categorical variables, n, %**	n	%	N	%
Women	3442	48.93	579	62.33
Age group				
65-<75 y/o	3780	53.73	165	17.76
75-<85	2743	38.99	419	45.10
> = 85	512	7.28	345	37.14
Body Mass Index (BMI)*				
< = 18	156	2.22	36	3.88
18<BMI< = 24	3204	45.54	392	42.20
24<BMI< = 30	2611	37.11	241	25.94
>30	301	4.28	35	3.77
Education years				
0	2427	34.50	561	60.39
1–6	2710	38.52	245	26.37
7–12	1338	19.02	90	9.69
>12	560	7.96	33	3.55
Marital status				
Married and live with spouse	4698	66.78	413	44.46
Widow or widower	2004	28.49	467	50.27
Others	333	4.73	49	5.27
Life style and habit				
Smoking	1436	20.41	128	13.78
Drinking	929	13.21	71	7.64
Chewing betel nut	347	4.93	28	3.01
Exercise				
Rare exercise	1955	27.79	654	70.47
Exercise sometimes	1762	25.05	173	18.64
Regular exercise	3191	45.36	101	10.88
Social activity				
Rare social activity	4376	62.20	808	86.98
Social activity sometimes	1507	21.42	80	8.61
Regular social activity	1152	16.38	41	4.41
Night sleep				
Good sleep	4208	59.82	389	41.87
Occasional poor sleep	1665	23.67	286	30.79
Often insomnia	553	7.86	129	13.89
Always insomnia	609	8.66	125	13.46
Afternoon nap habit				
No nap	1779	25.29	187	20.13
Occasional taking nap	1429	20.31	192	20.67
Taking nap everyday	3827	54.40	550	59.20
Comorbidities				
Hypertension	3512	50.91	513	57.90
Diabetes mellitus	1376	19.89	283	32.05
Cerebrovascular disease	251	3.59	188	20.77

*There were 988 missing data on BMI category.

Table 2 shows the association between each lifestyle factor and marital status with dementia using univariate logistic regression analyses. Associated factors with decreased odds include exercise, social activity, smoking, drinking, chewing betel nut and adequate night sleep. Widow or widower status and taking naps have increased odds of dementia.

**Table 2:** Crude ORs of the risk factors for dementia using univariate logistic regression analyses.

Variables	Crude ORs	*p* value	95% CI
Marital status (married and live with spouse as reference)			
Widow or widower	2.65	<0.01	2.30–3.06
Others	1.67	<0.01	1.22–2.30
Lifestyle			
Smoking	0.62	<0.01	0.51–0.76
Drinking	0.54	<0.01	0.42–0.70
Red wine	0.50	0.10	0.22–1.14
Beer	0.53	0.07	0.27–1.04
Sorghum	0.42	<0.01	0.24–0.73
Rice wine	0.59	0.17	0.27–1.27
Other	0.37	<0.01	0.20–0.70
Chewing betel nut	0.60	0.01	0.41–0.89
Exercise (rare exercise as reference)			
Exercise sometimes	0.29	<0.01	0.25–0.35
Regular exercise	0.09	<0.01	0.08–0.12
Social activity (rare social activity as reference)			
Social activity sometimes	0.29	<0.01	0.23–0.36
Regular social activity	0.19	<0.01	0.14–0.27
Night sleep (always insomnia as reference)			
Good sleep	0.45	<0.01	0.36–0.56
Occasional poor sleep	0.84	0.13	0.67–1.05
Often insomnia	1.14	0.36	0.87–1.49
Afternoon nap habit (no nap as reference)			
Occasional taking nap	1.28	0.02	1.03–1.58
Taking nap everyday	1.37	<0.01	1.15–1.63

CI: confidence interval; ORs: odds ratios.

After adjusting for gender, age and educational level (model 1, Table 3), associated factors with decreased odds include exercise, social engagement, and adequate night sleep, whereas widow/widower status and taking afternoon naps showed increased odds. We also adjusted for body mass index and comorbidities including hypertension, diabetes mellitus and history of cerebrovascular disease (model 2, Table 3). The results remained consistent with model 1. After full model adjustment for factors in model 1 and 2, as well as lifestyle factors and marital status (model 3, Table 3), exercise, social engagement, and adequate night sleep remained significant association factors dcreased odds for dementia. In contrast, widow/widower status and afternoon nap habit still showed increased odds for dementia.

**Table 3:** Adjusted ORs for association of dementia regarding marital status and lifestyle.

Variables	Model 1		Model 2		Model 3	
	ORs (95% CI)	*p* value	ORs (95% CI)	*p* value	ORs (95% CI)	*p* value
Marital status (married and live with spouse as reference)		<0.01		<0.01		<0.01
Widow or widower	1.38 (1.17–1.62)		1.46 (1.20–1.79)		1.42 (1.15–1.77)	
Others	1.49 (1.06–2.10)		1.23 (0.77–1.94)		1.20 (0.73–1.96)	
Lifestyle						
Smoking	0.88 (0.70–1.11)	0.28	0.73 (0.55–0.97)	0.03	0.73 (0.52–1.01)	0.06
Drinking Chewing betel nut	0.79 (0.59–1.04)	0.09	0.72 (0.51–1.01)	0.06	0.79 (0.54–1.17)	0.24
	1.11 (0.73–1.70)	0.61	0.97 (0.57–1.65)	0.90	1.04 (0.58–1.87)	0.89
Exercise (rare exercise as reference)		<0.01		<0.01		<0.01
exercise sometimes	0.32 (0.26–0.38)		0.36 (0.28–0.45)		0.41 (0.32–0.51)	
regular exercise	0.12 (0.09–0.15)		0.11 (0.08–0.15)		0.12 (0.09–0.16)	
Social engagement (rare engagement as reference)		<0.01		<0.01		<0.01
social activity sometimes	0.35 (0.27–0.44)		0.43 (0.32–0.57)		0.57 (0.42–0.77)	
regular social activity	0.25 (0.18–0.35)		0.38 (0.27–0.54)		0.53 (0.36–0.77)	
Night sleep (always insomnia as reference)		<0.01		<0.01		<0.01
good sleep	0.47 (0.37–0.60)		0.53 (0.39–0.72)		0.55 (0.39–0.76)	
occasional poor sleep	0.77 (0.60–0.99)		0.98 (0.72–1.35)		1.02 (0.73–1.44)	
often insomnia	1.00 (0.74–0.36)		1.04 (0.71–1.54)		1.00 (0.66–1.52)	
Afternoon nap habit (no nap as reference)		<0.01		<0.01		<0.01
occasional taking nap	1.19 (0.94–1.50)		1.35 (1.01–1.79)		1.39 (1.03–1.89)	
taking nap everyday	1.17 (0.97–1.41)		1.18 (0.92–1.50)		1.33 (1.02–1.72)	

Model 1 indicates model adjusted for age, gender and education level

Model 2 indicates model 1 plus adjustment for body mass index and comorbidities including hypertension, diabetes mellitus and history of cerebrovascular disease

Model 3 indicates model 2 plus adjustment for marital status and lifestyle including smoking, drinking, chewing betel nut, exercise, social engagement, night sleep, afternoon nap habit

CI: confidence interval; ORs: odds ratio.

Compared to people married and living with a spouse, the adjusted OR of dementia in people with widow or widower status was 1.42 (95% CI = 1.15–1.77). Compared to people who rarely exercised, the OR decreased to 0.12 (95%CI = 0.09–0.16) in those who regularly exercised. Compared to people who rarely had social activities, the OR was 0.53 (95% CI = 0.42–0.77) in people who had regular social activities. Compared to people who always had insomnia, the OR of dementia was 0.55 (95% CI = 0.39–0.76) in people with adequate sleep. Compared to people without afternoon naps, the OR of dementia 1.33 (95% CI = 1.03–1.89) in people who took a nap daily. No other significant associations between dementia and other variables, including smoking, drinking or chewing betel nuts. The Pearson's chi-squared statistic showed low Cramér's V, which indicated low association between each variable in this multiple logistic regression model.

## Discussion

In this nationwide population-based study, our results provide preliminary evidence that exercise, social engagement and adequate night sleep possibly have protective effects on dementia, whereas widow/widower status and afternoon nap habit might increase the odds. To the best of our knowledge, this is the first and only large-scale, nationwide epidemiology study, with a detailed random sampling plan of a geographic area, investigating dementia and its associated factors, including various daily lifestyle habits and marital states in Taiwan. In our study, all participants underwent a detailed assessment to make a reliable diagnosis of dementia, and the criteria were based on the core clinical criteria from the NIA-AA, which is one of the principle diagnostic guidelines for dementia research. These demographic data were extensively collected by an in-person, face-to-face interview to establish correct recording customized to the culture of this region and the lifestyles of Taiwanese or Asian people. Moreover, we also investigated marital status, which has not been well studied.

The results of this study showed that exercise is a significant association factor that decreases odds for dementia, which is consistent with other cross-sectional case-controlled or prospective cohort or meta-analysis studies in other countries.[11] Our study further noted that, after full adjustment of variable lifestyle factors, exercise still showed a decreased odds for dementia, whether “regular exercise” or “exercise sometimes”. This finding is encouraging for elderly people starting regular physical activity even modest amounts could benefit to reduce odds for dementia. This is an important strength of this report that in a cultural background of Taiwan that 45.4% of the elderly people do regular physical activity (at least once per week) in modest amounts.

Many studies have focused on the association between cognition and social activities in healthy people or patients with mild cognitive impairment and have shown that social activities and social support are correlated with better cognitive performance.[12–15]. In this study, we found that social activities at least once per week could decrease the risk of dementia. In Taiwanese society compared to western countries, many people go to “temples” instead of “churches” due to a different religion and “chat in a park” instead of “going to a party”. Additionally, the participants in this study performed “Tai-chi” more than going to the gym. Our results are encouraging because people can benefit from various types of social activities in various cultural and religious areas.

The association between sleep disturbance and cognitive function has attracted substantial interest of many researchers and clinicians in recent years. Multiple cross-sectional or prospective studies have revealed that sleep problems, such as insomnia, excessive daytime sleepiness, prolonged sleep time, sleep-disordered breathing, or increased daytime napping, may be correlated with cognitive decline.[16–19] The pathophysiological evidence for sleep problems contributing to cognitive impairment or dementia due to Alzheimer’s disease (AD) have been mainly demonstrated by animal models or biomarkers and functional image studies in humans. Sleep deprivation can lead to increased amyloid deposition in the brain and therefore accelerate neurodegeneration process. However, degeneration of brain regions for sleep promotion due to amyloid pathology can disturb sleep conditions.[20] Chronic insomnia is defined as having problems in falling asleep, maintaining sleep, early awakening or poor sleep quality for more than one month. However, the association between insomnia and cognitive decline remains controversial^26^. This debate may be due to different insomnia measurements, definitions and scales. In our study, we recorded the frequency of insomnia to define the severity of their sleep problem reported by family or caregivers and found that “adequate sleep” is a significant association factor that dcreaeses odds for dementia. To clarify these associations, we may need to further investigate the total sleep duration and to quantify daytime sleepiness precisely.

Our study revealed that napping is associated with a higher risk of dementia (either napping occasionally or daily). By regression analysis, the factor of nap became significant after adding the variables of exercise, social engagement and night sleep in the model. This means exercise, social engagement and night sleep may influence the factor of nap to become an independent factor. People who usually have their daytime full of exercise and social activities may not have time to develop the habit of nap. On the other hand, aged people with poor night sleep may appear inactive in the daytime and need nap to refresh. Another study showed that napping for more than 60 minutes is associated with AD morbidity among APOE**ε**4 carriers.[21] In contrast, some evidence suggests that napping is beneficial for cognitive performance but the amount varies according to the napping frequency and duration.[22, 23] A cross-sectional study revealed an inverted U-shape correlation between the frequency of napping and cognitive performance with the most benefit with 1–2 naps per week.[24] Another prospective study showed that daytime napping is associated with a lower risk of cognitive decline in a follow-up of 2 and 10 years, but the effect is attenuated if the napping is more than 60 minutes.[25] Our results need further investigation about nap duration, which may contribute to the negative cognition effect. Additionally, a napping habit may be due to night sleep fragmentation, poor sleep quality, and daytime hypersomnolence, which resulted in less social activity engagement and depressed mood, which are related to increased risk of dementia.[26]

Losing a spouse is certainly a stressful event and is one of the leading environmental risk factors of depression. Depressive mood or depression has been proved to be associated with cognitive performance and may increase risk of developing dementia in the future.[27] As a consequence, losing a spouse in mid-life or late life may increase the risk of developing cognitive decline, which is suggested by a longitudinal follow-up study.[28] Living alone or remaining unmarried status is also a risk of subsequent cognitive deterioration, as reported by previous studies.[29, 30] Our study also suggests that widows or widowers are at a higher risk of dementia among Taiwanese people.

There are some limitations in this study. First, we did not further separate various types of exercise and quantified the intensity and amount of exercise in this study, which may have had different strengths of association or causal relations with cognition.[31] Second, although various social activities were included in “social engagement”, we did not further identify which type of social activity with positive association to cognition. Third, the investigation questionnaire was based on report of the non-demented participant or observation and report of the proxy. This is indeed a global impression from collapse of time periods probably across decades of life. The informants were their clear selves or their family principal caretakers, usually their spouses, sons or daughters or in-laws who logically have the best knowledge about the cognitive-impaired participants. The history was taken from the participant or a knowledgeable informant, who provided at least 10 hours of weekly direct care for the patient with dementia. The lifestyle questionnaire has been applied in several different studies using clinical probands on the Taiwanese elderly population, which have received clinical or genetic validation.[32, 33] Fourth, people in the early stage of dementia may have subtle behavior changes, such as decreased social engagement due to lack of spontaneity and declined social skills, that informants might not have noted or might consider the subtle behavior change as a usual lifestyle habit rather than an underlying dementing process. We could not define when the past habit started, ended or changed before the onset of dementia or whether the habit change was due to the deterioration of dementia especially on those patients with degenerative dementia which takes a very insidious onset and slow progression course. Therefore, the observational bias and limitation from the cross-sectional design might result in a reversal of causality in terms of social activity. This is the limitation of all cross-sectional studies and even in some longitudinal studies with follow-up periods of only a few years (for example 3–5 years). The detection of changes of lifestyle habits and disentanglement of causal relationship probably could be tackled only by a prospective and very long-term (decades) cohort. This is beyond the scope of this report.

## Conclusions

Our research demonstrated that the risk of dementia was significantly associated with widow /widower status and the habit of afternoon napping. Habitual exercise even in modest amounts, regular social activities and adequate nighttime sleep protected against dementia in Taiwanese people. Our study provides evidence for future longitudinal studies to clarify the causal relationship and to estimate the scale of the impact of lifestyle factors on the progression of cognitive decline. The associations between marital status and sleep (including napping) and dementia, which are much less explored in previous studies, warrant special attention.
